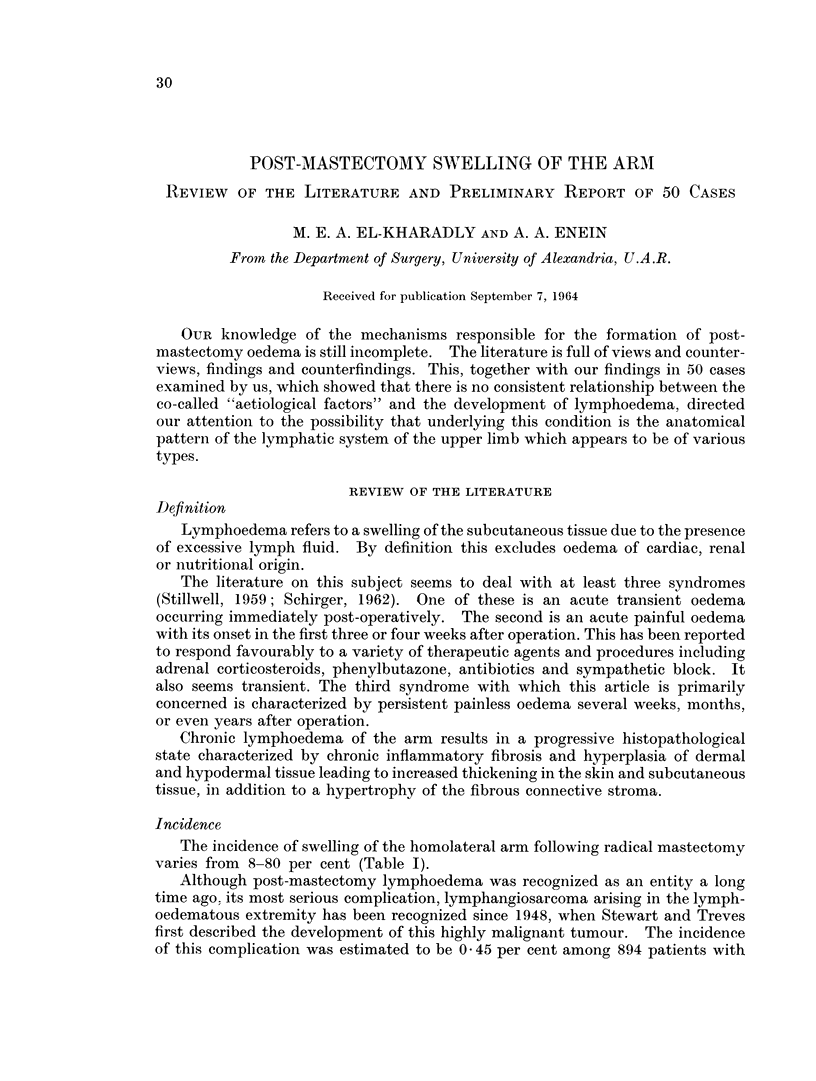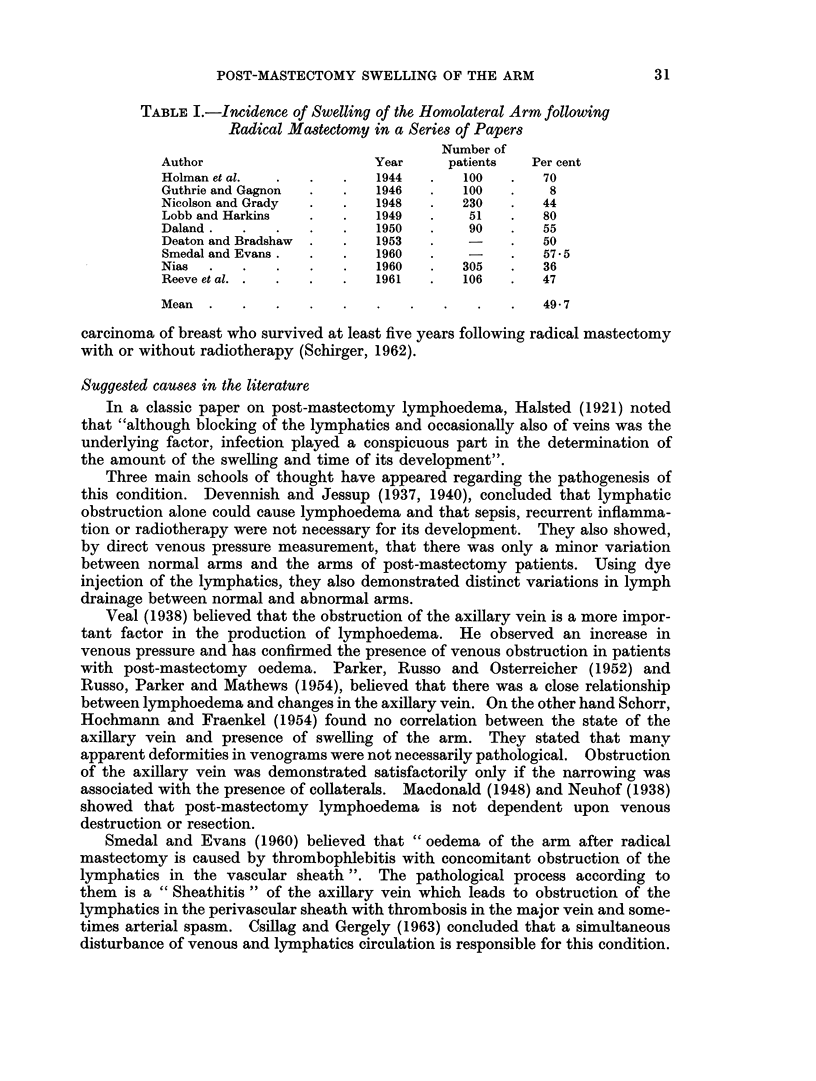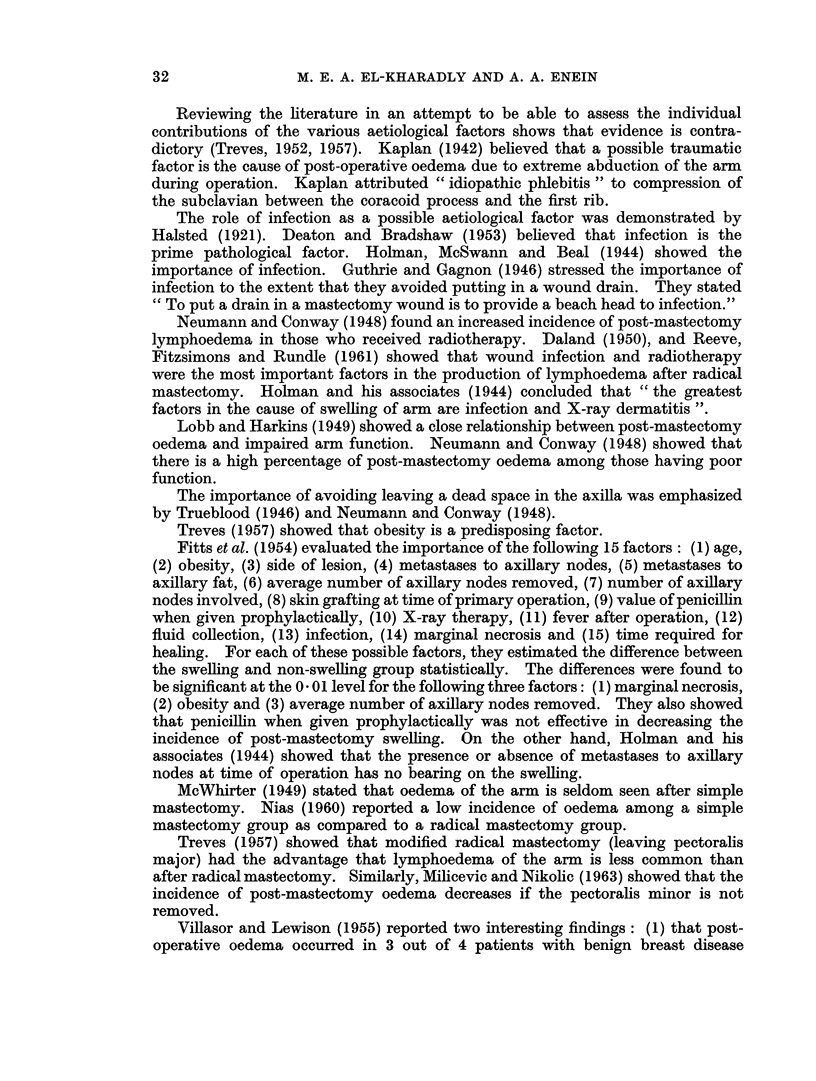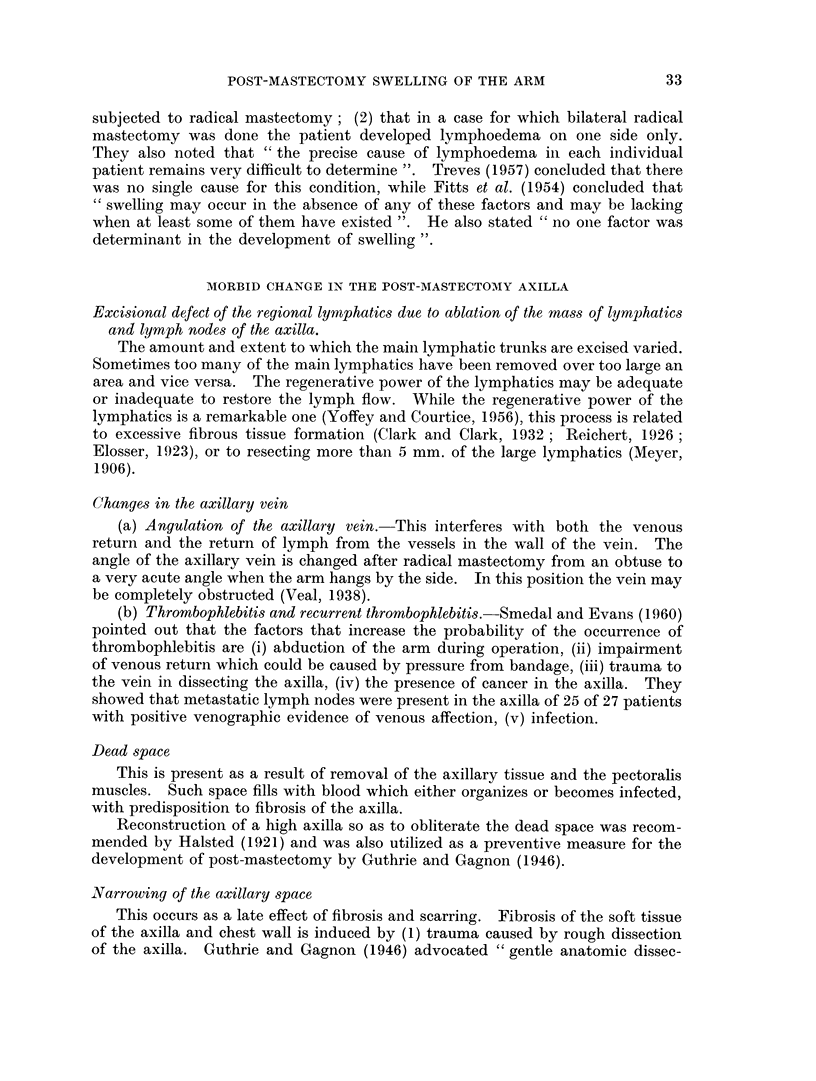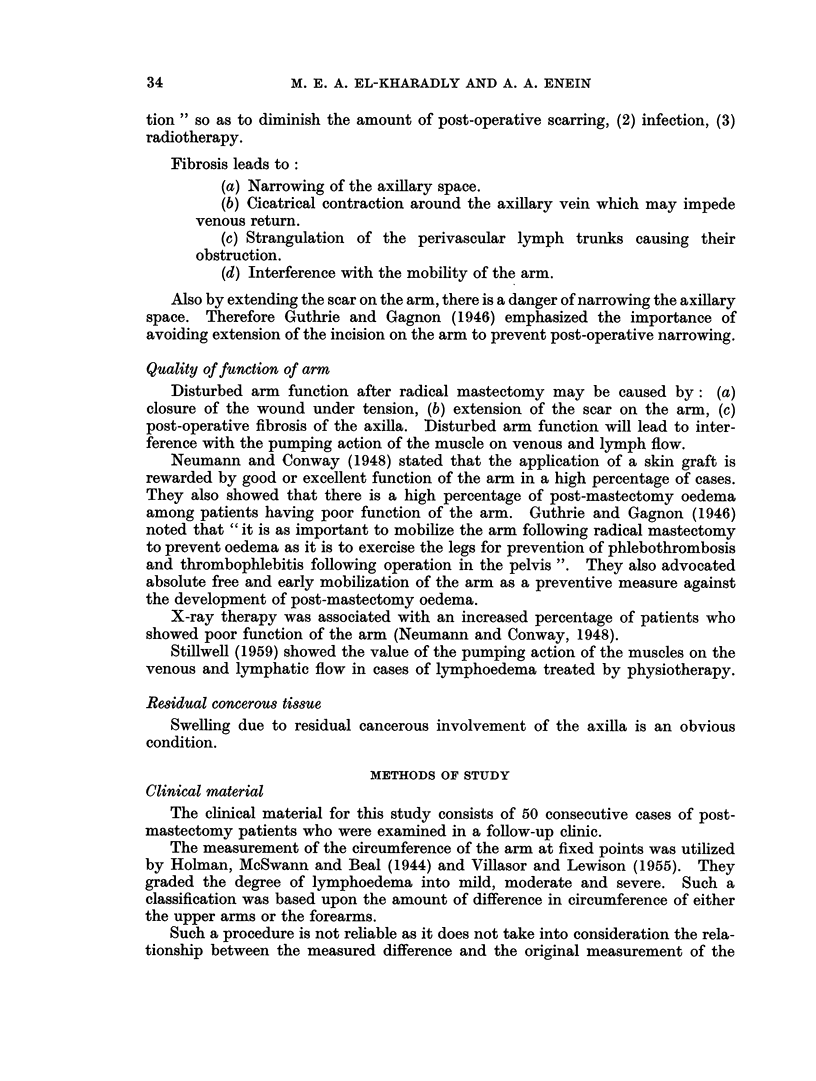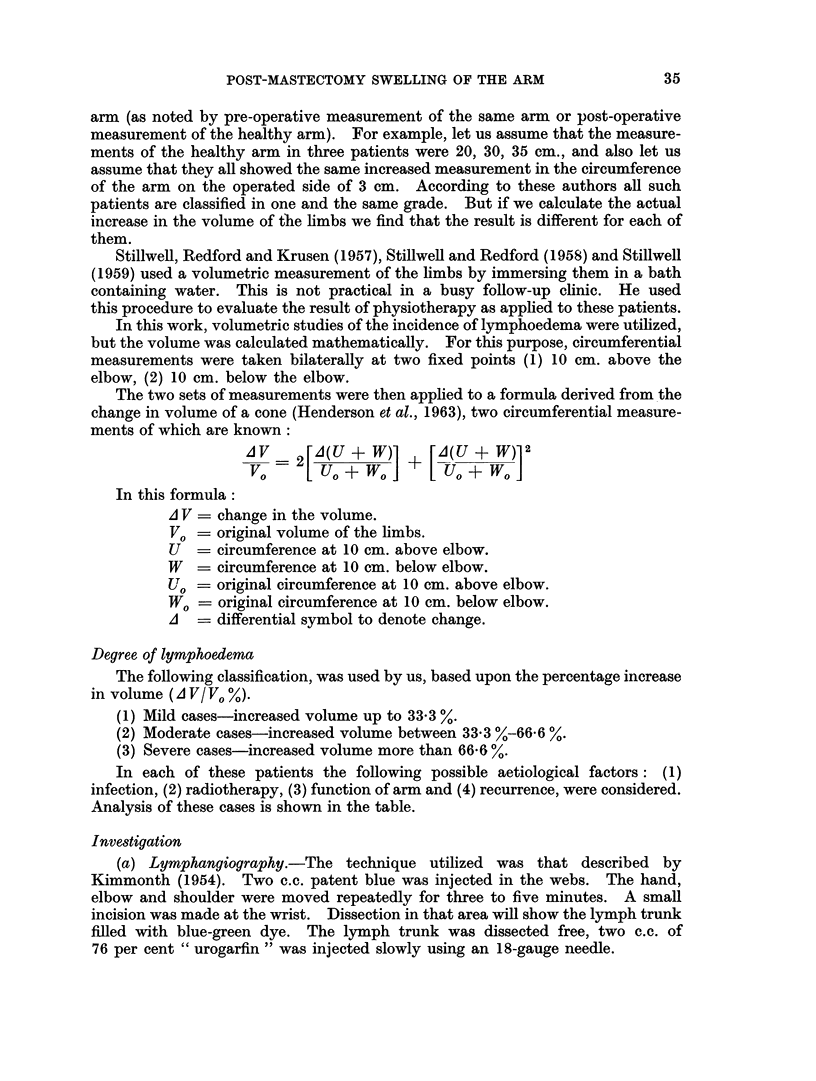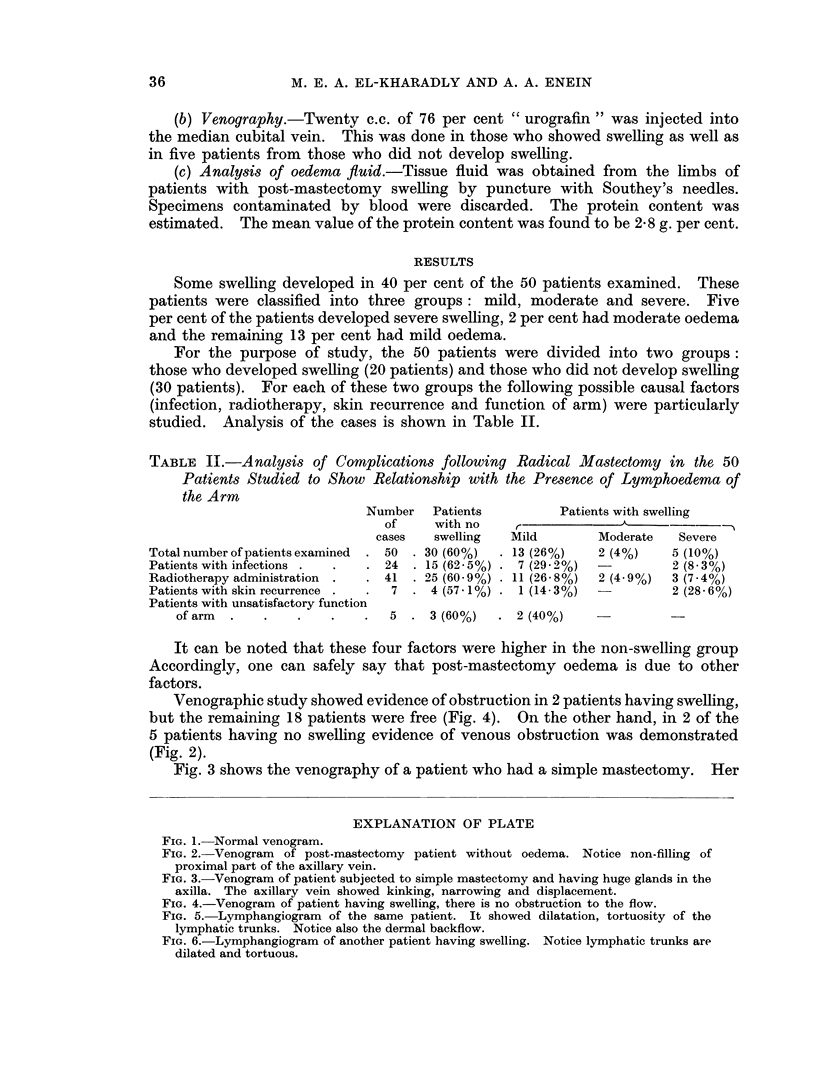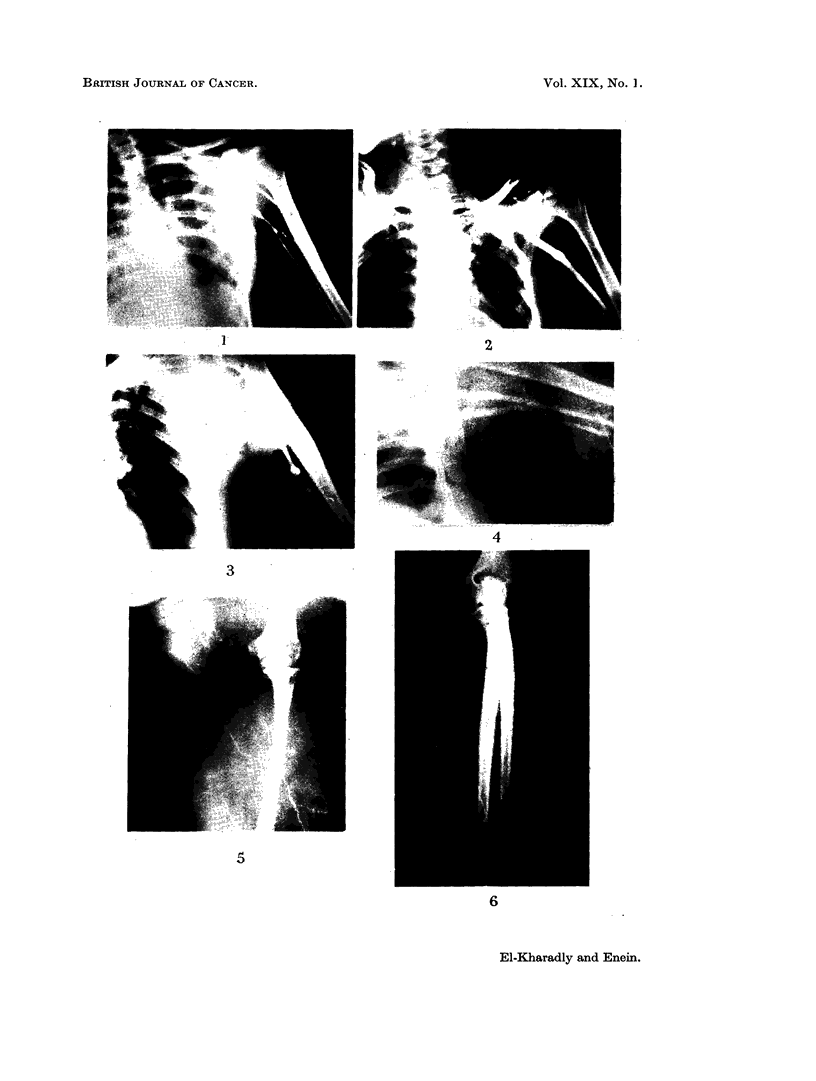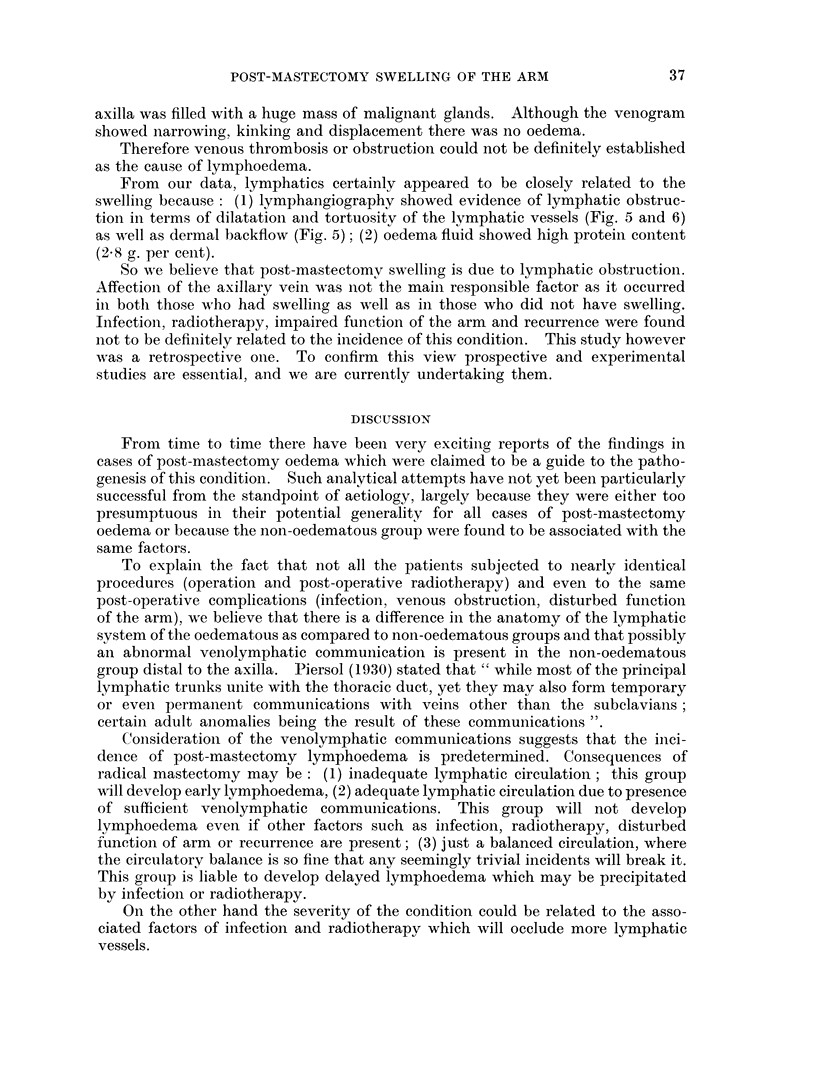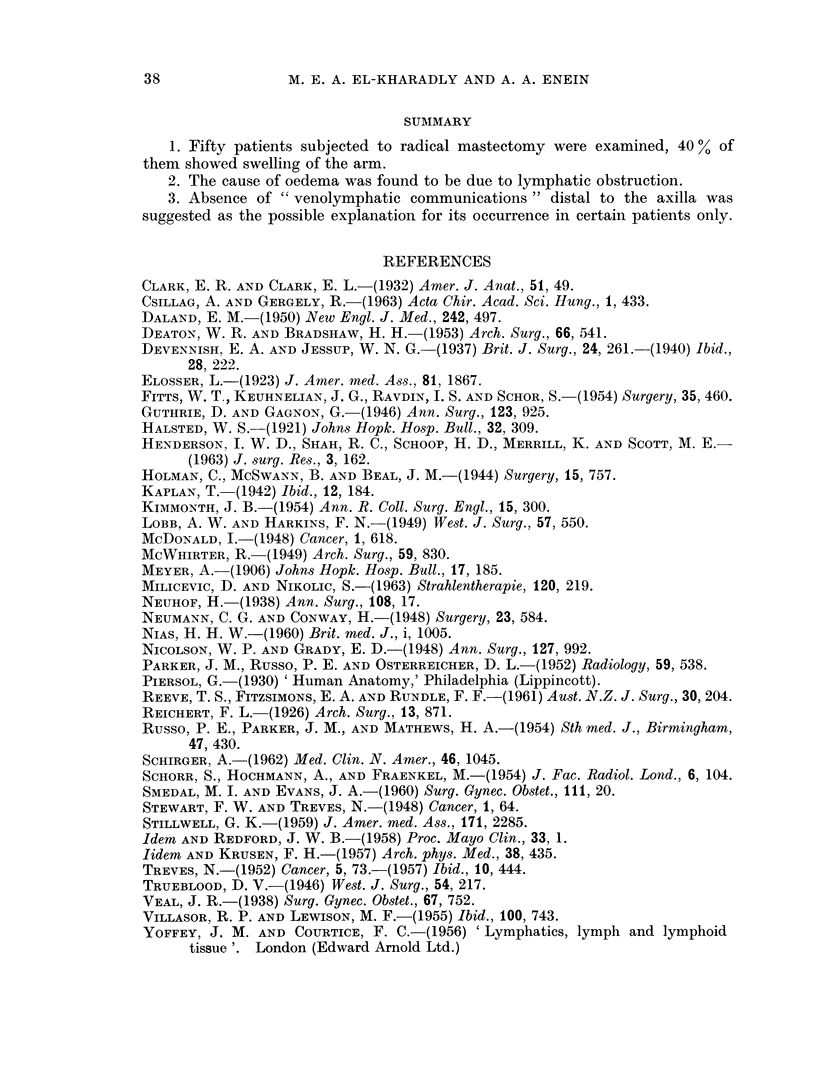# Post-Mastectomy Swelling of the Arm

**DOI:** 10.1038/bjc.1965.4

**Published:** 1965-03

**Authors:** M. E. A. El-Kharadly, A. A. Enein

## Abstract

**Images:**


### POST-MASTECTOMY SWELLING OF THE ARM

### Review of the Literature and Preliminary Report of 50 Cases

M. E. A. EL-KHARADLY and A. A. ENEIN

*From the Department of Surgery, University of Alexandria, U.A.R.*

Received for publication September 7, 1964

Our knowledge of the mechanisms responsible for the formation of post-mastectomy oedema is still incomplete. The literature is full of views and counter-views, findings and counterfindings. This, together with our findings in 50 cases examined by us, which showed that there is no consistent relationship between the co-called "aetiological factors" and the development of lymphoedema, directed our attention to the possibility that underlying this condition is the anatomical pattern of the lymphatic system of the upper limb which appears to be of various types.

### REVIEW OF THE LITERATURE

*Definition*

Lymphoedema refers to a swelling of the subcutaneous tissue due to the presence of excessive lymph fluid. By definition this excludes oedema of cardiac, renal or nutritional origin.

The literature on this subject seems to deal with at least three syndromes (Stillwell, 1959; Schirger, 1962). One of these is an acute transient oedema occurring immediately post-operatively. The second is an acute painful oedema with its onset in the first three or four weeks after operation. This has been reported to respond favourably to a variety of therapeutic agents and procedures including adrenal corticosteroids, phenylbutazone, antibiotics and sympathetic block. It also seems transient. The third syndrome with which this article is primarily concerned is characterized by persistent painless oedema several weeks, months, or even years after operation.

Chronic lymphoedema of the arm results in a progressive histopathological state characterized by chronic inflammatory fibrosis and hyperplasia of dermal and hypodermal tissue leading to increased thickening in the skin and subcutaneous tissue, in addition to a hypertrophy of the fibrous connective stroma.

*Incidence*

The incidence of swelling of the homolateral arm following radical mastectomy varies from 8–80 per cent (Table I).

Although post-mastectomy lymphoedema was recognized as an entity a long time ago, its most serious complication, lymphangiosarcoma arising in the lymph-oedematous extremity has been recognized since 1948, when Stewart and Treves first described the development of this highly malignant tumour. The incidence of this complication was estimated to be 0·45 per cent among 894 patients with

TABLE I.—*Incidence of Swelling of the Homolateral Arm following Radical Mastectomy in a Series of Papers*

| Author | Year | Number of patients | Per cent |
|---|---|---|---|
| Holman *et al.* | 1944 | 100 | 70 |
| Guthrie and Gagnon | 1946 | 100 | 8 |
| Nicolson and Grady | 1948 | 230 | 44 |
| Lobb and Harkins | 1949 | 51 | 80 |
| Daland | 1950 | 90 | 55 |
| Deaton and Bradshaw | 1953 | — | 50 |
| Smedal and Evans | 1960 | — | 57·5 |
| Nias | 1960 | 305 | 36 |
| Reeve *et al.* | 1961 | 106 | 47 |
| Mean | | | 49·7 |

carcinoma of breast who survived at least five years following radical mastectomy with or without radiotherapy (Schirger, 1962).

*Suggested causes in the literature*

In a classic paper on post-mastectomy lymphoedema, Halsted (1921) noted that "although blocking of the lymphatics and occasionally also of veins was the underlying factor, infection played a conspicuous part in the determination of the amount of the swelling and time of its development".

Three main schools of thought have appeared regarding the pathogenesis of this condition. Devennish and Jessup (1937, 1940), concluded that lymphatic obstruction alone could cause lymphoedema and that sepsis, recurrent inflammation or radiotherapy were not necessary for its development. They also showed, by direct venous pressure measurement, that there was only a minor variation between normal arms and the arms of post-mastectomy patients. Using dye injection of the lymphatics, they also demonstrated distinct variations in lymph drainage between normal and abnormal arms.

Veal (1938) believed that the obstruction of the axillary vein is a more important factor in the production of lymphoedema. He observed an increase in venous pressure and has confirmed the presence of venous obstruction in patients with post-mastectomy oedema. Parker, Russo and Osterreicher (1952) and Russo, Parker and Mathews (1954), believed that there was a close relationship between lymphoedema and changes in the axillary vein. On the other hand Schorr, Hochmann and Fraenkel (1954) found no correlation between the state of the axillary vein and presence of swelling of the arm. They stated that many apparent deformities in venograms were not necessarily pathological. Obstruction of the axillary vein was demonstrated satisfactorily only if the narrowing was associated with the presence of collaterals. Macdonald (1948) and Neuhof (1938) showed that post-mastectomy lymphoedema is not dependent upon venous destruction or resection.

Smedal and Evans (1960) believed that " oedema of the arm after radical mastectomy is caused by thrombophlebitis with concomitant obstruction of the lymphatics in the vascular sheath ". The pathological process according to them is a " Sheathitis " of the axillary vein which leads to obstruction of the lymphatics in the perivascular sheath with thrombosis in the major vein and sometimes arterial spasm. Csillag and Gergely (1963) concluded that a simultaneous disturbance of venous and lymphatics circulation is responsible for this condition.

Reviewing the literature in an attempt to be able to assess the individual contributions of the various aetiological factors shows that evidence is contradictory (Treves, 1952, 1957). Kaplan (1942) believed that a possible traumatic factor is the cause of post-operative oedema due to extreme abduction of the arm during operation. Kaplan attributed " idiopathic phlebitis " to compression of the subclavian between the coracoid process and the first rib.

The role of infection as a possible aetiological factor was demonstrated by Halsted (1921). Deaton and Bradshaw (1953) believed that infection is the prime pathological factor. Holman, McSwann and Beal (1944) showed the importance of infection. Guthrie and Gagnon (1946) stressed the importance of infection to the extent that they avoided putting in a wound drain. They stated " To put a drain in a mastectomy wound is to provide a beach head to infection."

Neumann and Conway (1948) found an increased incidence of post-mastectomy lymphoedema in those who received radiotherapy. Daland (1950), and Reeve, Fitzsimons and Rundle (1961) showed that wound infection and radiotherapy were the most important factors in the production of lymphoedema after radical mastectomy. Holman and his associates (1944) concluded that " the greatest factors in the cause of swelling of arm are infection and X-ray dermatitis ".

Lobb and Harkins (1949) showed a close relationship between post-mastectomy oedema and impaired arm function. Neumann and Conway (1948) showed that there is a high percentage of post-mastectomy oedema among those having poor function.

The importance of avoiding leaving a dead space in the axilla was emphasized by Trueblood (1946) and Neumann and Conway (1948).

Treves (1957) showed that obesity is a predisposing factor.

Fitts *et al.* (1954) evaluated the importance of the following 15 factors : (1) age, (2) obesity, (3) side of lesion, (4) metastases to axillary nodes, (5) metastases to axillary fat, (6) average number of axillary nodes removed, (7) number of axillary nodes involved, (8) skin grafting at time of primary operation, (9) value of penicillin when given prophylactically, (10) X-ray therapy, (11) fever after operation, (12) fluid collection, (13) infection, (14) marginal necrosis and (15) time required for healing. For each of these possible factors, they estimated the difference between the swelling and non-swelling group statistically. The differences were found to be significant at the 0·01 level for the following three factors: (1) marginal necrosis, (2) obesity and (3) average number of axillary nodes removed. They also showed that penicillin when given prophylactically was not effective in decreasing the incidence of post-mastectomy swelling. On the other hand, Holman and his associates (1944) showed that the presence or absence of metastases to axillary nodes at time of operation has no bearing on the swelling.

McWhirter (1949) stated that oedema of the arm is seldom seen after simple mastectomy. Nias (1960) reported a low incidence of oedema among a simple mastectomy group as compared to a radical mastectomy group.

Treves (1957) showed that modified radical mastectomy (leaving pectoralis major) had the advantage that lymphoedema of the arm is less common than after radical mastectomy. Similarly, Milicevic and Nikolic (1963) showed that the incidence of post-mastectomy oedema decreases if the pectoralis minor is not removed.

Villasor and Lewison (1955) reported two interesting findings : (1) that post-operative oedema occurred in 3 out of 4 patients with benign breast disease subjected to radical mastectomy ; (2) that in a case for which bilateral radical mastectomy was done the patient developed lymphoedema on one side only. They also noted that " the precise cause of lymphoedema in each individual patient remains very difficult to determine ". Treves (1957) concluded that there was no single cause for this condition, while Fitts *et al.* (1954) concluded that " swelling may occur in the absence of any of these factors and may be lacking when at least some of them have existed ". He also stated " no one factor was determinant in the development of swelling ".

### MORBID CHANGE IN THE POST-MASTECTOMY AXILLA

*Excisional defect of the regional lymphatics due to ablation of the mass of lymphatics and lymph nodes of the axilla.*

The amount and extent to which the main lymphatic trunks are excised varied. Sometimes too many of the main lymphatics have been removed over too large an area and vice versa. The regenerative power of the lymphatics may be adequate or inadequate to restore the lymph flow. While the regenerative power of the lymphatics is a remarkable one (Yoffey and Courtice, 1956), this process is related to excessive fibrous tissue formation (Clark and Clark, 1932 ; Reichert, 1926 ; Elosser, 1923), or to resecting more than 5 mm. of the large lymphatics (Meyer, 1906).

*Changes in the axillary vein*

(a) *Angulation of the axillary vein.*—This interferes with both the venous return and the return of lymph from the vessels in the wall of the vein. The angle of the axillary vein is changed after radical mastectomy from an obtuse to a very acute angle when the arm hangs by the side. In this position the vein may be completely obstructed (Veal, 1938).

(b) *Thrombophlebitis and recurrent thrombophlebitis.*—Smedal and Evans (1960) pointed out that the factors that increase the probability of the occurrence of thrombophlebitis are (i) abduction of the arm during operation, (ii) impairment of venous return which could be caused by pressure from bandage, (iii) trauma to the vein in dissecting the axilla, (iv) the presence of cancer in the axilla. They showed that metastatic lymph nodes were present in the axilla of 25 of 27 patients with positive venographic evidence of venous affection, (v) infection.

*Dead space*

This is present as a result of removal of the axillary tissue and the pectoralis muscles. Such space fills with blood which either organizes or becomes infected, with predisposition to fibrosis of the axilla.

Reconstruction of a high axilla so as to obliterate the dead space was recommended by Halsted (1921) and was also utilized as a preventive measure for the development of post-mastectomy by Guthrie and Gagnon (1946).

*Narrowing of the axillary space*

This occurs as a late effect of fibrosis and scarring. Fibrosis of the soft tissue of the axilla and chest wall is induced by (1) trauma caused by rough dissection of the axilla. Guthrie and Gagnon (1946) advocated " gentle anatomic dissection " so as to diminish the amount of post-operative scarring, (2) infection, (3) radiotherapy.

Fibrosis leads to :

(*a*) Narrowing of the axillary space.

(*b*) Cicatrical contraction around the axillary vein which may impede venous return.

(*c*) Strangulation of the perivascular lymph trunks causing their obstruction.

(*d*) Interference with the mobility of the arm.

Also by extending the scar on the arm, there is a danger of narrowing the axillary space. Therefore Guthrie and Gagnon (1946) emphasized the importance of avoiding extension of the incision on the arm to prevent post-operative narrowing.

*Quality of function of arm*

Disturbed arm function after radical mastectomy may be caused by: (*a*) closure of the wound under tension, (*b*) extension of the scar on the arm, (*c*) post-operative fibrosis of the axilla. Disturbed arm function will lead to interference with the pumping action of the muscle on venous and lymph flow.

Neumann and Conway (1948) stated that the application of a skin graft is rewarded by good or excellent function of the arm in a high percentage of cases. They also showed that there is a high percentage of post-mastectomy oedema among patients having poor function of the arm. Guthrie and Gagnon (1946) noted that "it is as important to mobilize the arm following radical mastectomy to prevent oedema as it is to exercise the legs for prevention of phlebothrombosis and thrombophlebitis following operation in the pelvis ". They also advocated absolute free and early mobilization of the arm as a preventive measure against the development of post-mastectomy oedema.

X-ray therapy was associated with an increased percentage of patients who showed poor function of the arm (Neumann and Conway, 1948).

Stillwell (1959) showed the value of the pumping action of the muscles on the venous and lymphatic flow in cases of lymphoedema treated by physiotherapy.

*Residual concerous tissue*

Swelling due to residual cancerous involvement of the axilla is an obvious condition.

### METHODS OF STUDY

*Clinical material*

The clinical material for this study consists of 50 consecutive cases of post-mastectomy patients who were examined in a follow-up clinic.

The measurement of the circumference of the arm at fixed points was utilized by Holman, McSwann and Beal (1944) and Villasor and Lewison (1955). They graded the degree of lymphoedema into mild, moderate and severe. Such a classification was based upon the amount of difference in circumference of either the upper arms or the forearms.

Such a procedure is not reliable as it does not take into consideration the relationship between the measured difference and the original measurement of the arm (as noted by pre-operative measurement of the same arm or post-operative measurement of the healthy arm). For example, let us assume that the measurements of the healthy arm in three patients were 20, 30, 35 cm., and also let us assume that they all showed the same increased measurement in the circumference of the arm on the operated side of 3 cm. According to these authors all such patients are classified in one and the same grade. But if we calculate the actual increase in the volume of the limbs we find that the result is different for each of them.

Stillwell, Redford and Krusen (1957), Stillwell and Redford (1958) and Stillwell (1959) used a volumetric measurement of the limbs by immersing them in a bath containing water. This is not practical in a busy follow-up clinic. He used this procedure to evaluate the result of physiotherapy as applied to these patients.

In this work, volumetric studies of the incidence of lymphoedema were utilized, but the volume was calculated mathematically. For this purpose, circumferential measurements were taken bilaterally at two fixed points (1) 10 cm. above the elbow, (2) 10 cm. below the elbow.

The two sets of measurements were then applied to a formula derived from the change in volume of a cone (Henderson *et al.*, 1963), two circumferential measurements of which are known :

$$\frac{\Delta V}{V_o} = 2\left[\frac{\Delta(U + W)}{U_o + W_o}\right] + \left[\frac{\Delta(U + W)}{U_o + W_o}\right]^2$$

In this formula :

$\Delta V$ = change in the volume.
$V_o$ = original volume of the limbs.
$U$ = circumference at 10 cm. above elbow.
$W$ = circumference at 10 cm. below elbow.
$U_o$ = original circumference at 10 cm. above elbow.
$W_o$ = original circumference at 10 cm. below elbow.
$\Delta$ = differential symbol to denote change.

*Degree of lymphoedema*

The following classification, was used by us, based upon the percentage increase in volume ($\Delta V/V_o$%).

(1) Mild cases—increased volume up to 33·3 %.
(2) Moderate cases—increased volume between 33·3 %–66·6 %.
(3) Severe cases—increased volume more than 66·6 %.

In each of these patients the following possible aetiological factors : (1) infection, (2) radiotherapy, (3) function of arm and (4) recurrence, were considered. Analysis of these cases is shown in the table.

*Investigation*

(*a*) *Lymphangiography.*—The technique utilized was that described by Kimmonth (1954). Two c.c. patent blue was injected in the webs. The hand, elbow and shoulder were moved repeatedly for three to five minutes. A small incision was made at the wrist. Dissection in that area will show the lymph trunk filled with blue-green dye. The lymph trunk was dissected free, two c.c. of 76 per cent " urogarfin " was injected slowly using an 18-gauge needle.

(*b*) *Venography.*—Twenty c.c. of 76 per cent " urografin " was injected into the median cubital vein. This was done in those who showed swelling as well as in five patients from those who did not develop swelling.

(*c*) *Analysis of oedema fluid.*—Tissue fluid was obtained from the limbs of patients with post-mastectomy swelling by puncture with Southey's needles. Specimens contaminated by blood were discarded. The protein content was estimated. The mean value of the protein content was found to be 2·8 g. per cent.

### RESULTS

Some swelling developed in 40 per cent of the 50 patients examined. These patients were classified into three groups : mild, moderate and severe. Five per cent of the patients developed severe swelling, 2 per cent had moderate oedema and the remaining 13 per cent had mild oedema.

For the purpose of study, the 50 patients were divided into two groups : those who developed swelling (20 patients) and those who did not develop swelling (30 patients). For each of these two groups the following possible causal factors (infection, radiotherapy, skin recurrence and function of arm) were particularly studied. Analysis of the cases is shown in Table II.

TABLE II.—*Analysis of Complications following Radical Mastectomy in the* 50 *Patients Studied to Show Relationship with the Presence of Lymphoedema of the Arm*

| | Number of cases | Patients with no swelling | Patients with swelling | | |
|---|---|---|---|---|---|
| | | | Mild | Moderate | Severe |
| Total number of patients examined | 50 | 30 (60%) | 13 (26%) | 2 (4%) | 5 (10%) |
| Patients with infections | 24 | 15 (62·5%) | 7 (29·2%) | — | 2 (8·3%) |
| Radiotherapy administration | 41 | 25 (60·9%) | 11 (26·8%) | 2 (4·9%) | 3 (7·4%) |
| Patients with skin recurrence | 7 | 4 (57·1%) | 1 (14·3%) | — | 2 (28·6%) |
| Patients with unsatisfactory function of arm | 5 | 3 (60%) | 2 (40%) | — | — |

It can be noted that these four factors were higher in the non-swelling group Accordingly, one can safely say that post-mastectomy oedema is due to other factors.

Venographic study showed evidence of obstruction in 2 patients having swelling, but the remaining 18 patients were free (Fig. 4). On the other hand, in 2 of the 5 patients having no swelling evidence of venous obstruction was demonstrated (Fig. 2).

Fig. 3 shows the venography of a patient who had a simple mastectomy. Her

---

### EXPLANATION OF PLATE

FIG. 1.—Normal venogram.

FIG. 2.—Venogram of post-mastectomy patient without oedema. Notice non-filling of proximal part of the axillary vein.

FIG. 3.—Venogram of patient subjected to simple mastectomy and having huge glands in the axilla. The axillary vein showed kinking, narrowing and displacement.

FIG. 4.—Venogram of patient having swelling, there is no obstruction to the flow.

FIG. 5.—Lymphangiogram of the same patient. It showed dilatation, tortuosity of the lymphatic trunks. Notice also the dermal backflow.

FIG. 6.—Lymphangiogram of another patient having swelling. Notice lymphatic trunks are dilated and tortuous.

1

2

3

4

5

6

El-Kharadly and Enein.

axilla was filled with a huge mass of malignant glands. Although the venogram showed narrowing, kinking and displacement there was no oedema.

Therefore venous thrombosis or obstruction could not be definitely established as the cause of lymphoedema.

From our data, lymphatics certainly appeared to be closely related to the swelling because : (1) lymphangiography showed evidence of lymphatic obstruction in terms of dilatation and tortuosity of the lymphatic vessels (Fig. 5 and 6) as well as dermal backflow (Fig. 5) ; (2) oedema fluid showed high protein content (2·8 g. per cent).

So we believe that post-mastectomy swelling is due to lymphatic obstruction. Affection of the axillary vein was not the main responsible factor as it occurred in both those who had swelling as well as in those who did not have swelling. Infection, radiotherapy, impaired function of the arm and recurrence were found not to be definitely related to the incidence of this condition. This study however was a retrospective one. To confirm this view prospective and experimental studies are essential, and we are currently undertaking them.

### DISCUSSION

From time to time there have been very exciting reports of the findings in cases of post-mastectomy oedema which were claimed to be a guide to the pathogenesis of this condition. Such analytical attempts have not yet been particularly successful from the standpoint of aetiology, largely because they were either too presumptuous in their potential generality for all cases of post-mastectomy oedema or because the non-oedematous group were found to be associated with the same factors.

To explain the fact that not all the patients subjected to nearly identical procedures (operation and post-operative radiotherapy) and even to the same post-operative complications (infection, venous obstruction, disturbed function of the arm), we believe that there is a difference in the anatomy of the lymphatic system of the oedematous as compared to non-oedematous groups and that possibly an abnormal venolymphatic communication is present in the non-oedematous group distal to the axilla. Piersol (1930) stated that " while most of the principal lymphatic trunks unite with the thoracic duct, yet they may also form temporary or even permanent communications with veins other than the subclavians ; certain adult anomalies being the result of these communications ".

Consideration of the venolymphatic communications suggests that the incidence of post-mastectomy lymphoedema is predetermined. Consequences of radical mastectomy may be : (1) inadequate lymphatic circulation ; this group will develop early lymphoedema, (2) adequate lymphatic circulation due to presence of sufficient venolymphatic communications. This group will not develop lymphoedema even if other factors such as infection, radiotherapy, disturbed function of arm or recurrence are present; (3) just a balanced circulation, where the circulatory balance is so fine that any seemingly trivial incidents will break it. This group is liable to develop delayed lymphoedema which may be precipitated by infection or radiotherapy.

On the other hand the severity of the condition could be related to the associated factors of infection and radiotherapy which will occlude more lymphatic vessels.

SUMMARY

1. Fifty patients subjected to radical mastectomy were examined, 40% of them showed swelling of the arm.

2. The cause of oedema was found to be due to lymphatic obstruction.

3. Absence of "venolymphatic communications" distal to the axilla was suggested as the possible explanation for its occurrence in certain patients only.

### REFERENCES

CLARK, E. R. AND CLARK, E. L.—(1932) *Amer. J. Anat.*, **51**, 49.

CSILLAG, A. AND GERGELY, R.—(1963) *Acta Chir. Acad. Sci. Hung.*, **1**, 433.

DALAND, E. M.—(1950) *New Engl. J. Med.*, **242**, 497.

DEATON, W. R. AND BRADSHAW, H. H.—(1953) *Arch. Surg.*, **66**, 541.

DEVENNISH, E. A. AND JESSUP, W. N. G.—(1937) *Brit. J. Surg.*, **24**, 261.—(1940) *Ibid.*, **28**, 222.

ELOSSER, L.—(1923) *J. Amer. med. Ass.*, **81**, 1867.

FITTS, W. T., KEUHNELIAN, J. G., RAVDIN, I. S. AND SCHOR, S.—(1954) *Surgery*, **35**, 460.

GUTHRIE, D. AND GAGNON, G.—(1946) *Ann. Surg.*, **123**, 925.

HALSTED, W. S.—(1921) *Johns Hopk. Hosp. Bull.*, **32**, 309.

HENDERSON, I. W. D., SHAH, R. C., SCHOOP, H. D., MERRILL, K. AND SCOTT, M. E.—(1963) *J. surg. Res.*, **3**, 162.

HOLMAN, C., MCSWANN, B. AND BEAL, J. M.—(1944) *Surgery*, **15**, 757.

KAPLAN, T.—(1942) *Ibid.*, **12**, 184.

KIMMONTH, J. B.—(1954) *Ann. R. Coll. Surg. Engl.*, **15**, 300.

LOBB, A. W. AND HARKINS, F. N.—(1949) *West. J. Surg.*, **57**, 550.

MCDONALD, I.—(1948) *Cancer*, **1**, 618.

MCWHIRTER, R.—(1949) *Arch. Surg.*, **59**, 830.

MEYER, A.—(1906) *Johns Hopk. Hosp. Bull.*, **17**, 185.

MILICEVIC, D. AND NIKOLIC, S.—(1963) *Strahlentherapie*, **120**, 219.

NEUHOF, H.—(1938) *Ann. Surg.*, **108**, 17.

NEUMANN, C. G. AND CONWAY, H.—(1948) *Surgery*, **23**, 584.

NIAS, H. H. W.—(1960) *Brit. med. J.*, i, 1005.

NICOLSON, W. P. AND GRADY, E. D.—(1948) *Ann. Surg.*, **127**, 992.

PARKER, J. M., RUSSO, P. E. AND OSTERREICHER, D. L.—(1952) *Radiology*, **59**, 538.

PIERSOL, G.—(1930) 'Human Anatomy,' Philadelphia (Lippincott).

REEVE, T. S., FITZSIMONS, E. A. AND RUNDLE, F. F.—(1961) *Aust. N.Z. J. Surg.*, **30**, 204.

REICHERT, F. L.—(1926) *Arch. Surg.*, **13**, 871.

RUSSO, P. E., PARKER, J. M., AND MATHEWS, H. A.—(1954) *Sth med. J., Birmingham*, **47**, 430.

SCHIRGER, A.—(1962) *Med. Clin. N. Amer.*, **46**, 1045.

SCHORR, S., HOCHMANN, A., AND FRAENKEL, M.—(1954) *J. Fac. Radiol. Lond.*, **6**, 104.

SMEDAL, M. I. AND EVANS, J. A.—(1960) *Surg. Gynec. Obstet.*, **111**, 20.

STEWART, F. W. AND TREVES, N.—(1948) *Cancer*, **1**, 64.

STILLWELL, G. K.—(1959) *J. Amer. med. Ass.*, **171**, 2285.

*Idem* AND REDFORD, J. W. B.—(1958) *Proc. Mayo Clin.*, **33**, 1.

*Iidem* AND KRUSEN, F. H.—(1957) *Arch. phys. Med.*, **38**, 435.

TREVES, N.—(1952) *Cancer*, **5**, 73.—(1957) *Ibid.*, **10**, 444.

TRUEBLOOD, D. V.—(1946) *West. J. Surg.*, **54**, 217.

VEAL, J. R.—(1938) *Surg. Gynec. Obstet.*, **67**, 752.

VILLASOR, R. P. AND LEWISON, M. F.—(1955) *Ibid.*, **100**, 743.

YOFFEY, J. M. AND COURTICE, F. C.—(1956) 'Lymphatics, lymph and lymphoid tissue'. London (Edward Arnold Ltd.)